# Unicentric form of Castleman´s disease, pitfalls of diagnosis and surgical treatment

**DOI:** 10.3389/fonc.2023.1057683

**Published:** 2023-01-30

**Authors:** Jiri Molacek, Vladislav Treska, Tomas Skalicky, Josef Vodicka, Jiri Ferda, Eva Ferdova, Jan Baxa, Christiana Mach, Alexandra Jungova, Michael Michal

**Affiliations:** ^1^ Faculty of Medicine in Pilsen, Charles University, Plzen, Czechia; ^2^ Department of Surgery, Faculty of Medicine in Pilsen, Charles University, Pilsen, Czechia; ^3^ Department of Imaging Methods, Faculty of Medicine in Pilsen, Charles University, Pilsen, Czechia; ^4^ Department of Hematology and Oncology, University Hospital in Pilsen, Plzen, Czechia

**Keywords:** Castleman disease, unicentric Castleman, surgical treatment, diagnosis, pitfalls

## Abstract

**Background:**

Castleman´s disease is an extremely rare heterogenous lymphoproliferative pathology with a mostly benign behavior. It is a localized or generalized lymph node enlargement of an unknown aetiology. Unicentric form is typically a slow-growing solitary mass occurring mostly in the mediastinum, abdominal cavity, retroperitoneum, pelvis and neck. Aetiology and pathogenesis of CD is probably diverse, varying in different types of this heterogeneous disease.

**Materials and Methods:**

Authors present a review of this issue based on their extensive experience. The aim is to summarize the crucial factors in the management of diagnostics and a surgical treatment of the unicentric form of Castleman´s disease. One of the key issues in the unicentric form is precise preoperative diagnostics and thus choosing the right surgical treatment strategy. Authors highlight pitfalls of the diagnosis and surgical treatment.

**Results:**

All histological types such as a hyaline vascular type, plasmacytic type and a mixed type are presented as well as options of surgical and conservative treatment. Differential diagnosis and malignant potential is discussed.

**Conclusion:**

Patients with Castleman´s disease should be treated in the high- volume centers, with a great experience in major surgical procedures as well as with preoperative imaging diagnostic techniques. Specialized pathologists and oncologists focusing on this issue are also absolutely necessary to avoid misdiagnosis. Only this complex approach can lead to excellent outcomes in patients with UCD.

## Introduction

Castleman´s disease (CD) is an extremely rare heterogenous lymphoproliferative disease with a mostly benign behavior which was described by Benjamin Castleman in 1950´s (1). It is a localized or generalized lymph node enlargement of an unknown aetiology. Multiple classifications have been described, however, CD may be divided into 3 major forms - unicentric (UCD), HHV-8-associated multicentric (HHV-8-MCD) and HHV-8-negative multicentric (HHV-8-negative MCD). UCD is typically a slow-growing solitary mass occurring mostly in the mediastinum, abdominal cavity, retroperitoneum, pelvis and neck. In such cases, surgery is the treatment of choice and has a curative potential. Nevertheless, further screening is recommended especially at specialized hematologic centers. In contrast, MCD affects multiple lymph nodes all over the body and these patients usually have severe symptoms and are treated by a hematooncologist usually after lymph node harvesting (2, 3). One of the key issues in UCD is precise preoperative diagnostics which is indispensable for choosing the right surgical treatment strategy. For various reasons, the diagnosis is finalized in some cases after a surgical removal of the mass. The aim of this paper is to summarize the crucial factors in the diagnostic process and the surgical treatment of the UCD.

## Unicentric form of Castleman´s disease

### Epidemiology

The unicentric form of Castleman disease is usually a minority in a wide spectrum of pathologies called Castleman disease (4–8). It is an affection of a single lymph node or multiple nodes in one location. The incidence and prevalence are not fully clear. The data available in the literature set the incidence at around 20 cases per million a year (9–12). The disease typically occurs in the 3^rd^-4^th^ decade and has no gender predilection. The aetiology of UCD remains unknown, although various hypotheses have been postulated.

### Histological types

**Figure 1:**
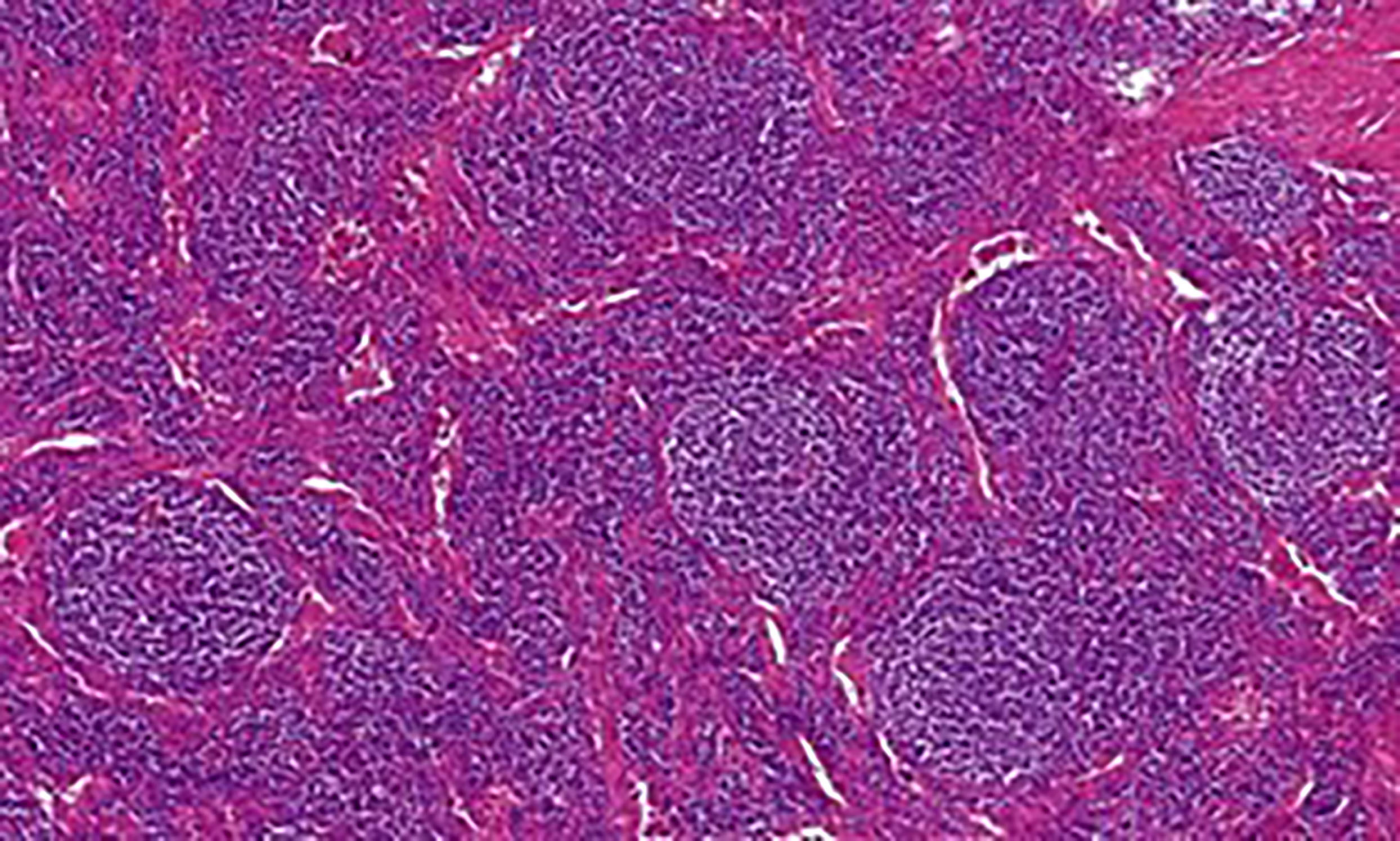
Castleman disease, hyaline-vascular type: Histology showed numerous enlarged follicles with abnormal germinal centers and with concentric layering of lymphocytes in the periphery of the follicles (onion-skin pattern of the mantle zone). Abnormal vascular proliferation and hyalinization was present within and in between the follicles.

**Figure 2:**
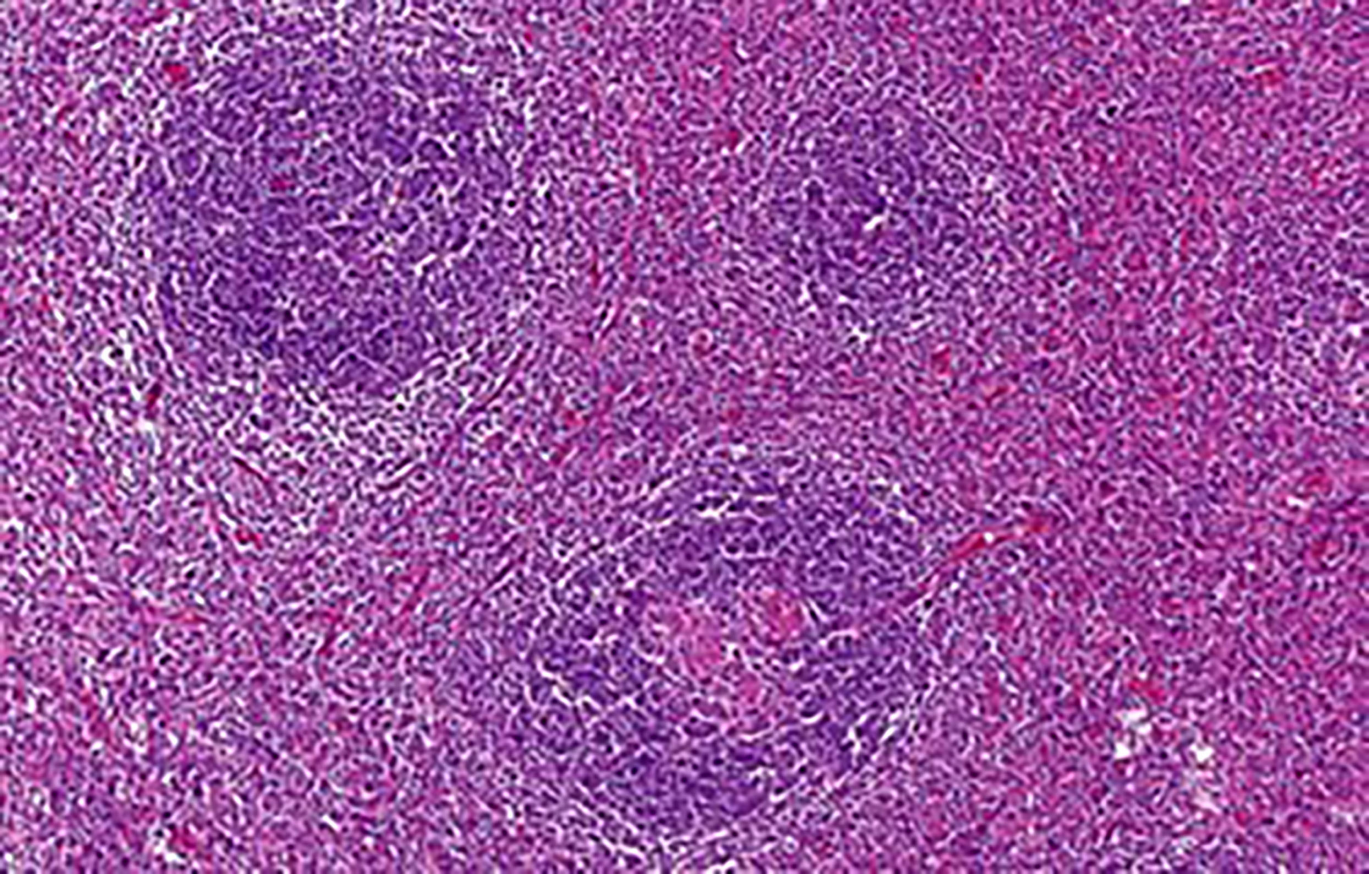
Some of the enlarged follicles were traversed by abnormal hyalinized vessels (lollipop sign). The interfollicular stroma was composed of a heterogeneous mixture of lymphocytes, plasma cells and occasional eosinophils.

**Figure 3:**
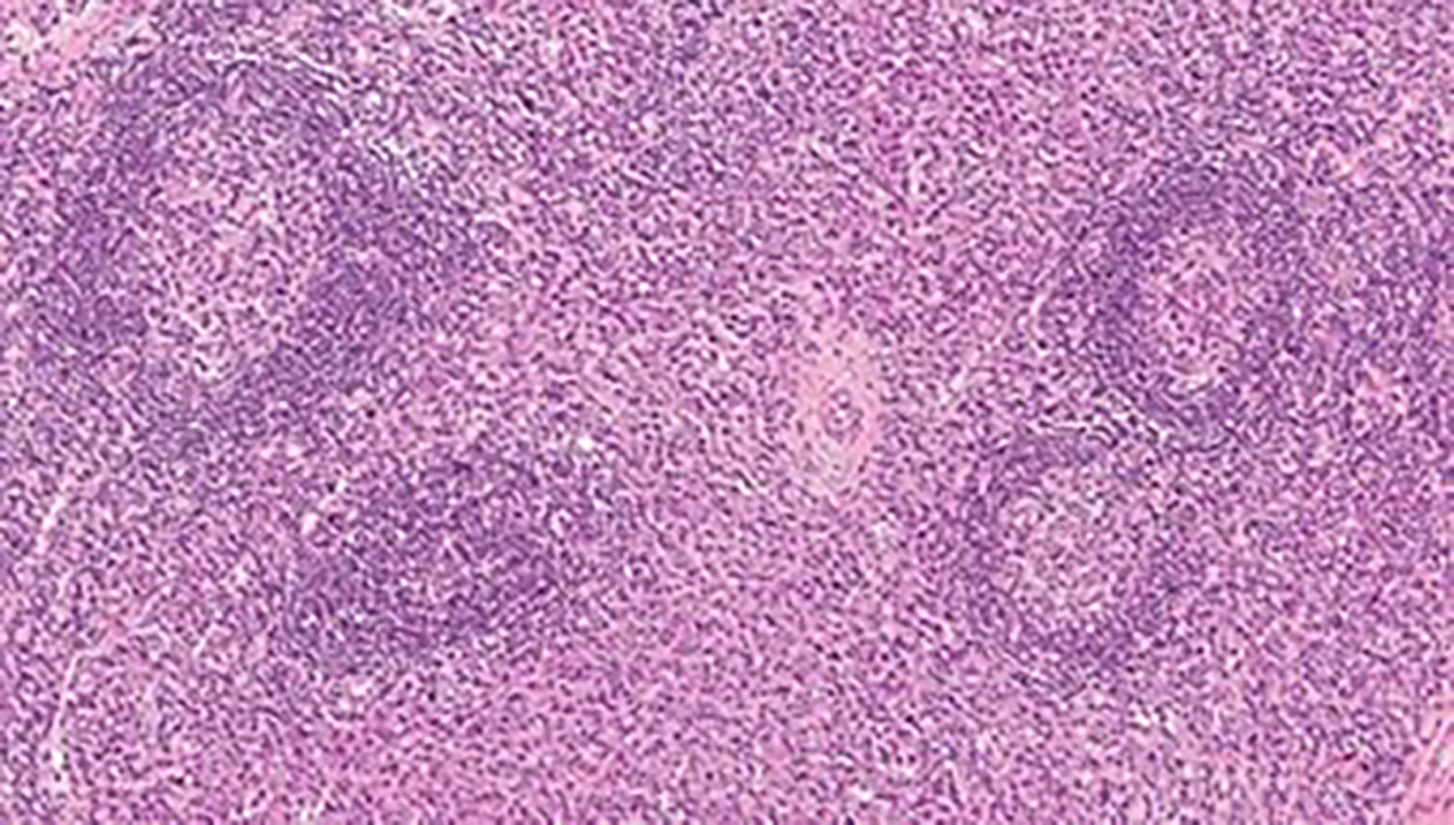
CD plasma cell type: numerous hyperplastic follicles with “onion skin” pattern in the mantle zone. The interfollicular area is almost exclusively composed of plasma cell infiltrate.

**Figure 4:**
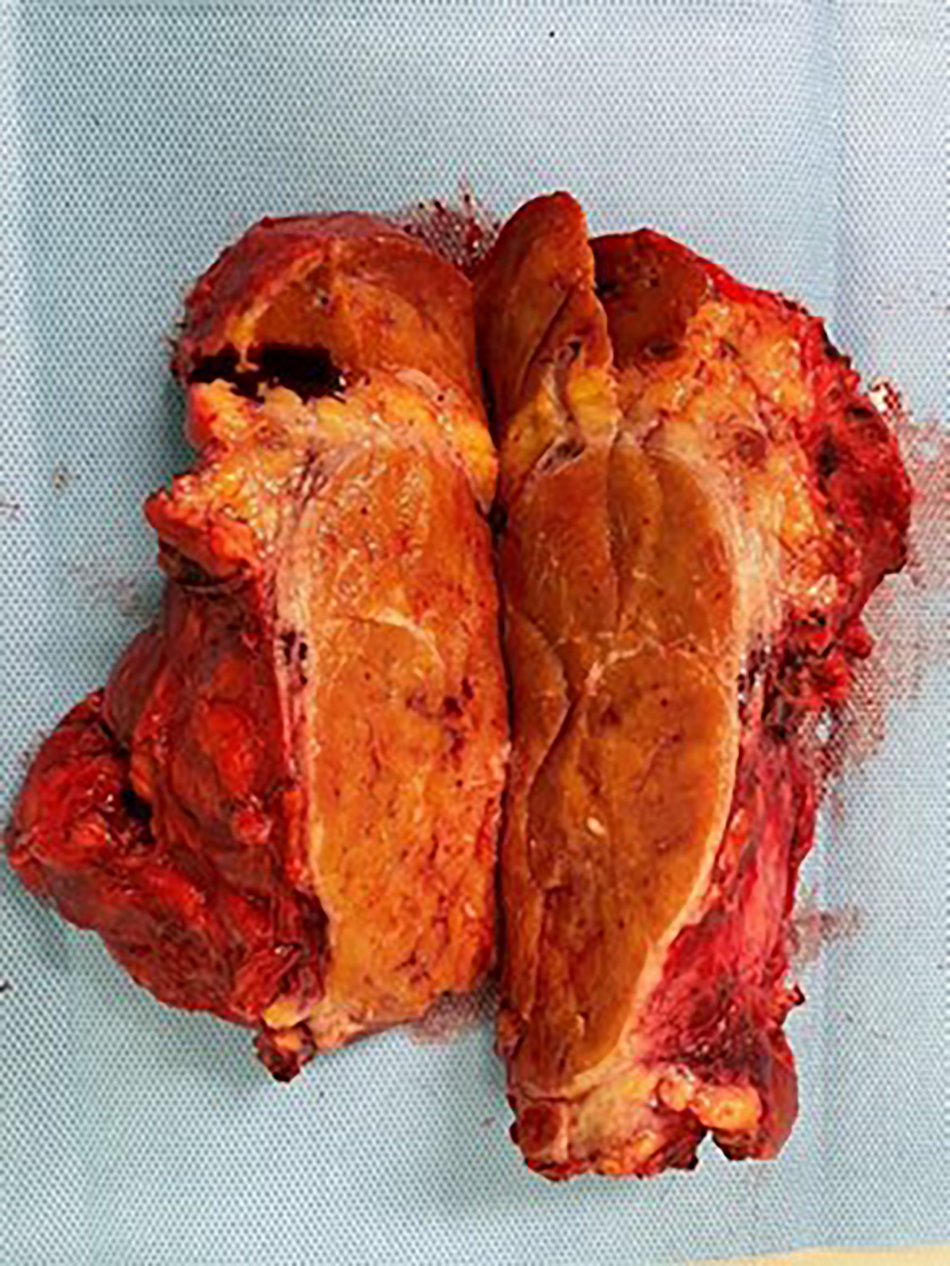
Typical fibrous capsule surrounding the lymph node in UCD.

According to the histopathological analysis, we can distinguish the following subtypes: a hyaline vascular type (HV) (**Figures 1**, **2**), a plasma cell type (PC) (**Figure 3**) and a mixed type. The HV type represents the most common form of UCD – about 70-80% (3, 8). Typically, it has a fibrous capsule surrounding the lymph node (see **Figure 4**).

### Symptoms

The course of the UCD is most often asymptomatic and many cases are found incidentally during a physical examination or imaging. Potential symptoms arise from the size of the mass and the compression of surrounding structures. Patients often discover palpable resistance on their own. Pain or discomfort are the most common complaints of patients. The severity of symptoms obviously increases with the size of the mass. Other symptoms include weight loss (more than 10% per 6 months), night sweats and subfebrile or febrile periods.

### Localization

**Figure 5:**
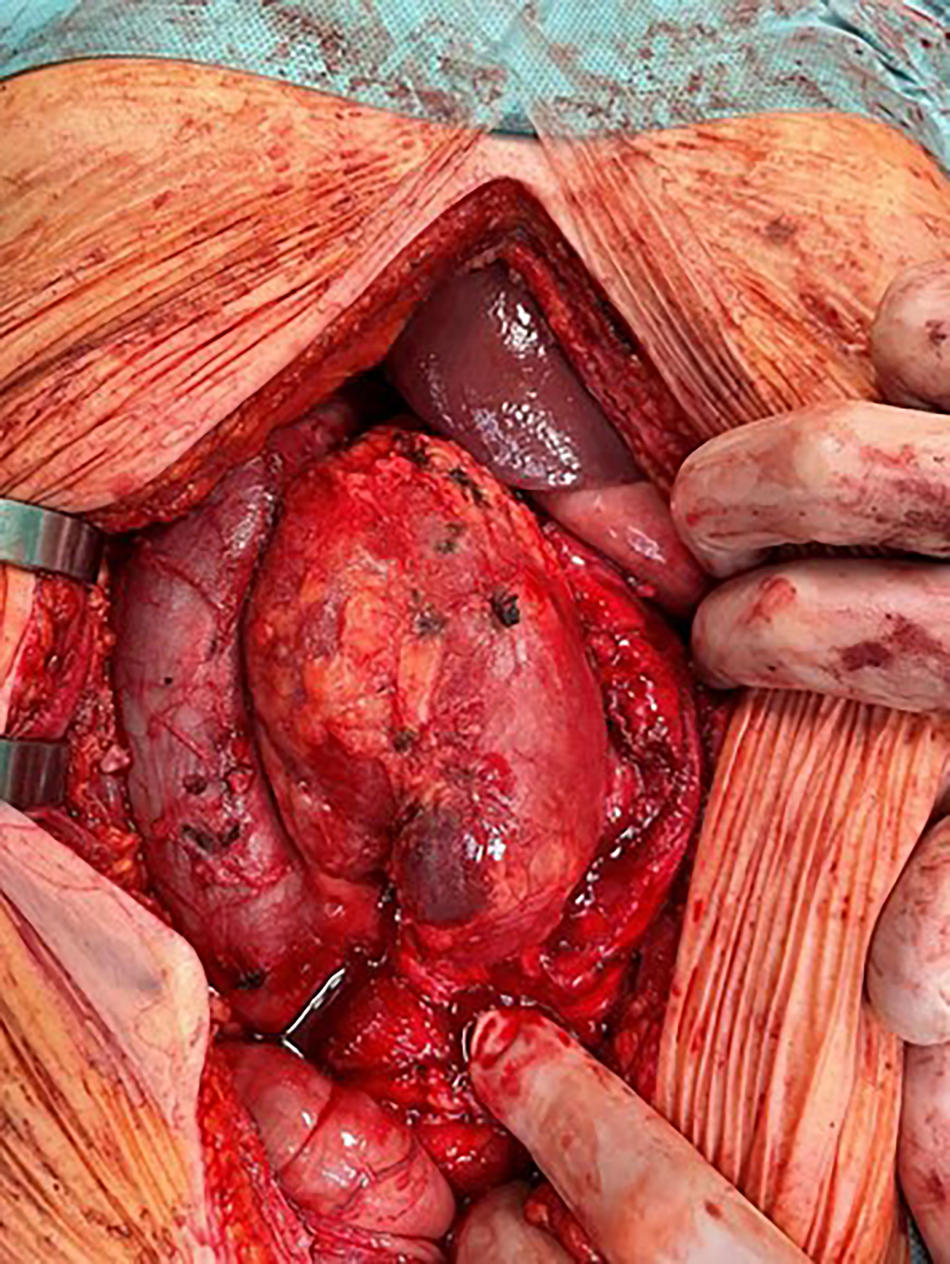
Unicentric CD in the retroperitoneal space between the aorta and inferior vena cava.

**Figure 6:**
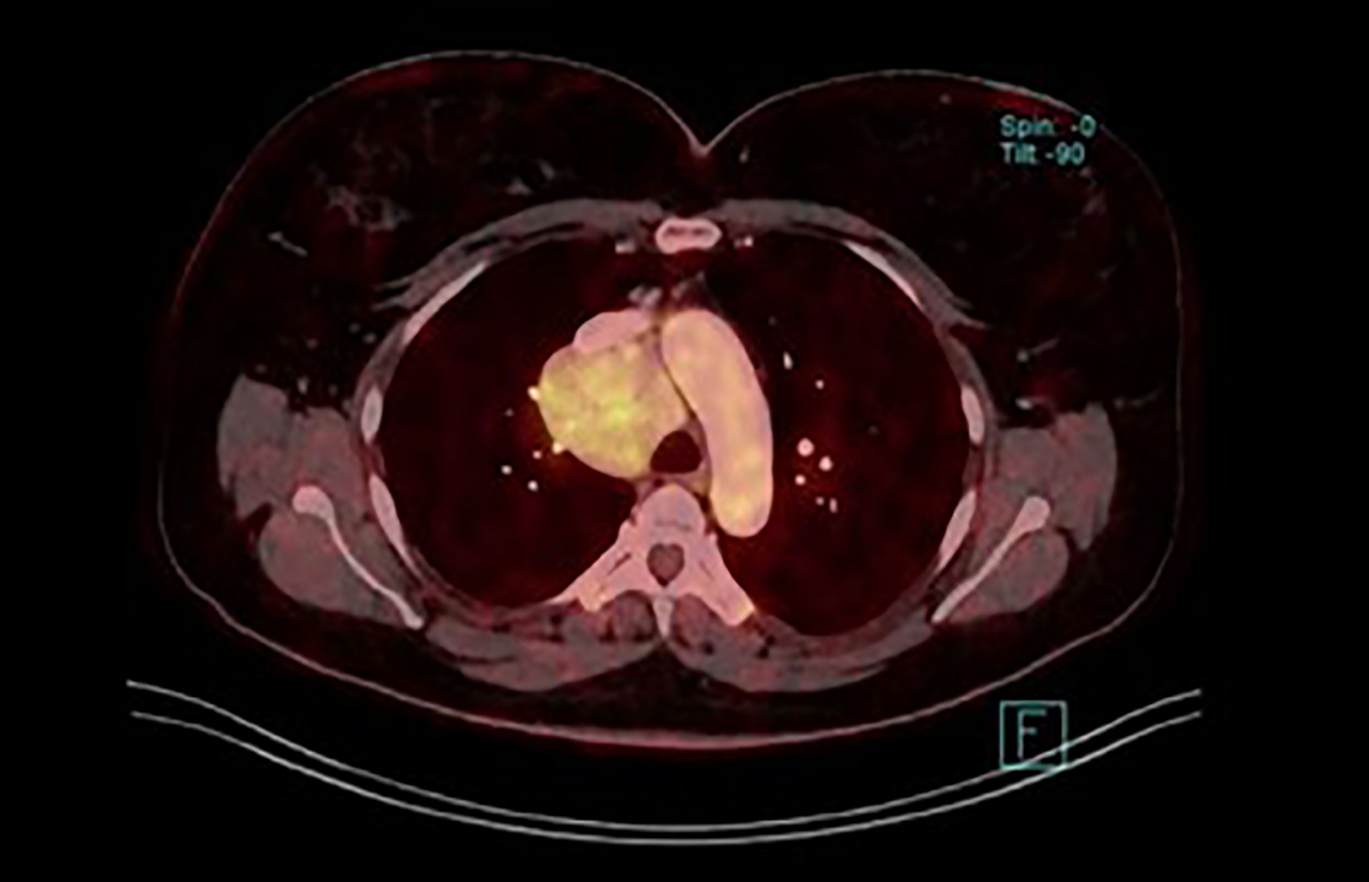
Unicentric CD in the mediastinum (fused image of the fluordeoxyglucose uptake and postcontrast CT from whole body PET/CT scan).

The most common location for UCD is the mediastinum, neck, abdominal cavity, retroperitoneum and pelvis. Axillary or inguinal region is affected less often (8, 13, 14). Cases in the retroperitoneum can often grow unnoticed for a long time and reach particularly large dimensions. A similar situation may occur in the mediastinum (**Figures 5**, **6**).

### Diagnostic methods

Imaging methods are the most crucial part of the diagnostic process, following the clinical examination. The ultrasound (US), computed tomography (CT), magnetic resonance (MRI) and also hybrid methods such as positron emission tomography with computed tomography (PET/CT) or magnetic resonance (PET/MRI) could be used for detection or localization of pathological lymph nodes or tumor mass and possibly for biopsy planning. Preoperative biopsy is very valuable, although it may not always be fully diagnostic. According to the available literature it remains a controversial issue (15) but we still believe that Fine Needle Aspiration Biopsy (FNAB) is very helpful.

**Figure 7:**
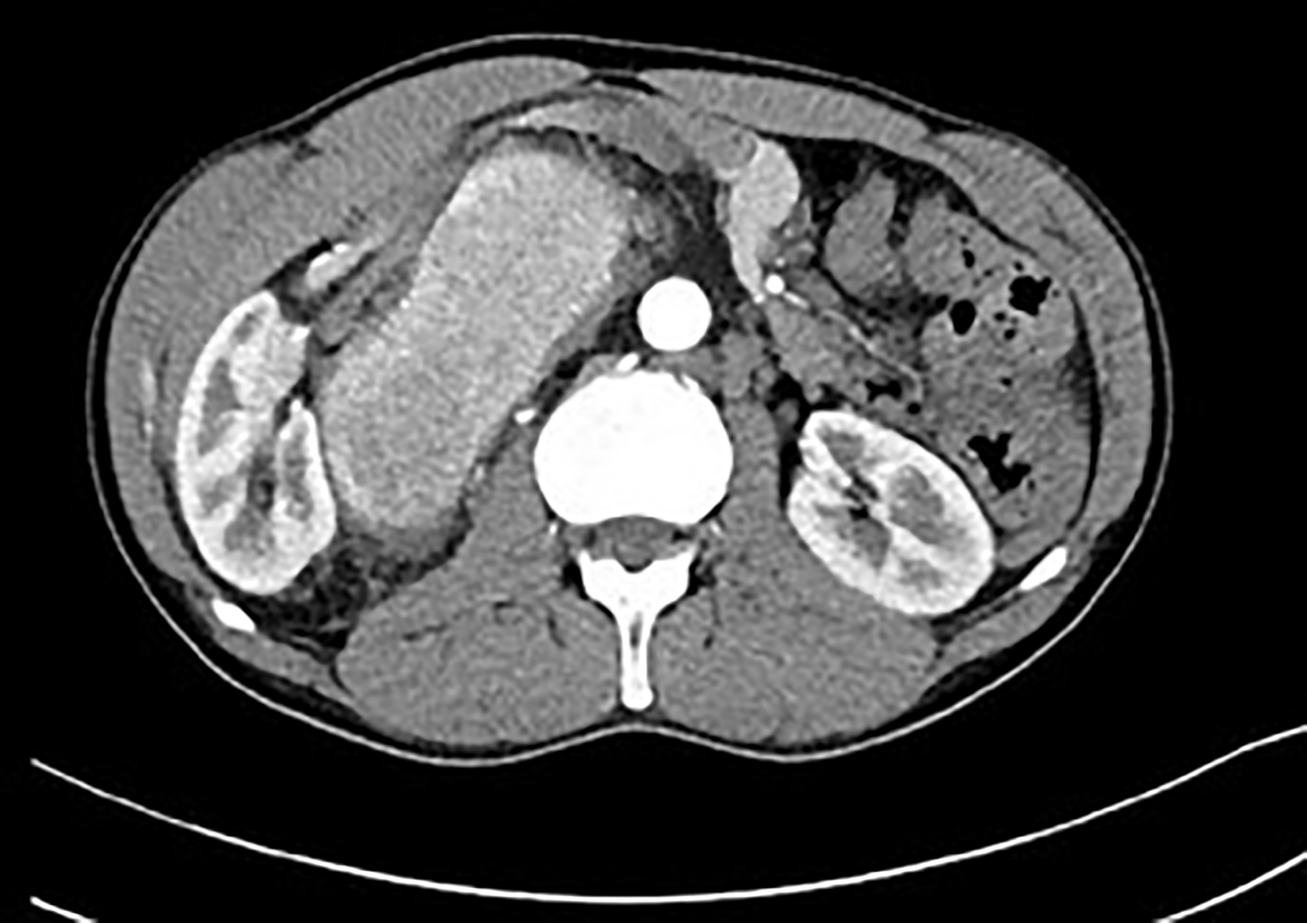
Postcontrast CT of unicentric form of Castleman disease. Markedly enhanced mass in right retroperitoneal space displacing right kidney.

The typical appearance of the angiofollicular hyperplasia of the lymph node or Castleman disease depends on the inner structure changes. The hyaline transformation of the hyperplastic vessels and the presence of plasma cells causes the hypoechogenic appearance on ultrasound, however these features do not allow to differentiate this disease from other lymphadenopathies. On the other hand, the hyperplasia of the vessels within the enlarged lymph node is the cause of the marked enhancement of the intravenously applied contrast agent both in computed tomography and magnetic resonance imaging (**Figure 7**). The Castleman disease appears as a well-defined and significantly enhancing mass displacing surrounding structures. The enhancement lasts during the venous phase due to the hyalinized structure of the vessels and the escape of contrast agent into the extravascular space leads to a decreased enhancement observed in late phases. This later decrease of enhancement could be used in the differential diagnosis with neuroendocrine tumors.

**Figure 8:**
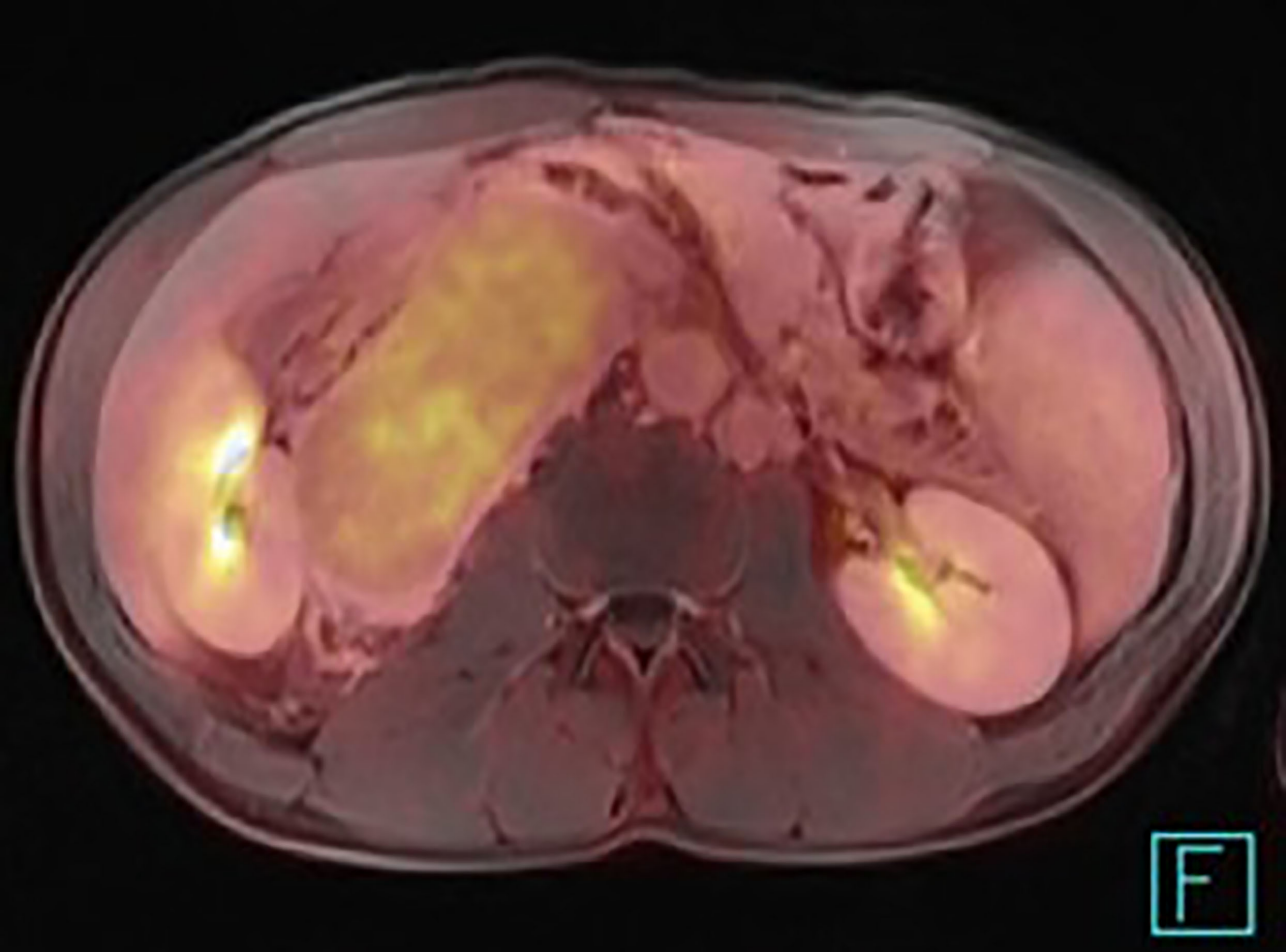
PET/MRI of the unicentric form of Castleman disease presenting a relatively low uptake of 18F-fluorodeoxyglucose.

**Figure 9:**
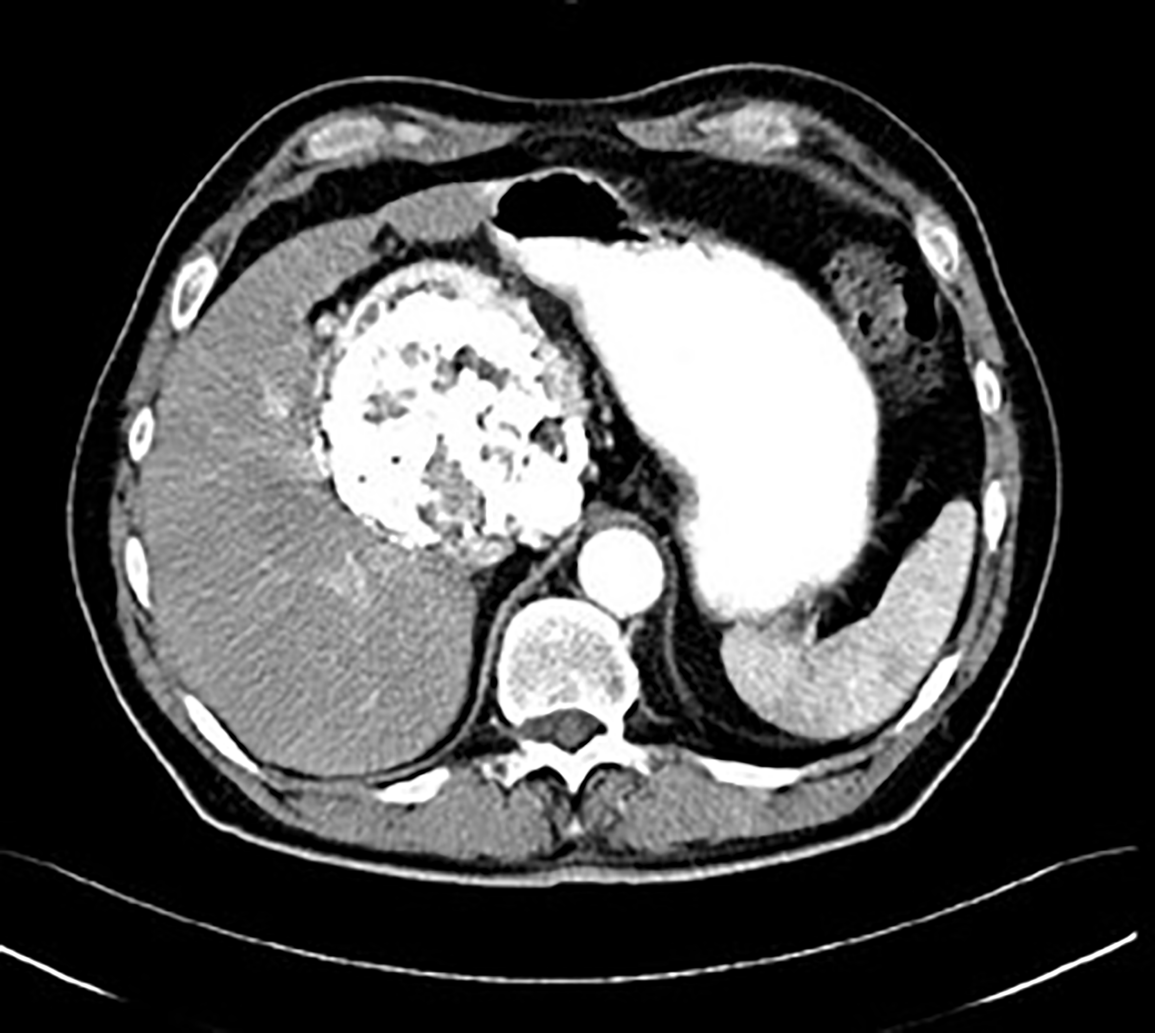
Postcontrast CT of unicentric form of Castleman disease - atypical finding of calcified mass in subhepatic space, histology: hyaline-vascular type.

When the positron emission tomography is used, 18F-fluorodeoxyglucose (FDG) is the optimal radiopharmaceutical agent (**Figure 8**). The level of glycolysis should be significantly lower than in e.g. poorly differentiated paragangliomas or more aggressive lymphoproliferative diseases. The differential diagnosis depends on the anatomic localition. The multicentric disease could be distinguished from other lymphoproliferative diseases by using the marked enhancement of the affected lymph nodes as well, but the important factor is the presence of the clinical and laboratory symptoms, which are absent in the unicentric form. Despite advanced imaging techniques, definitive preoperative diagnosis of the CD could be challenging. Some findings are atypical and may mislead the diagnostic procedure (**Figure 9**).

### Differential diagnosis

**Figure 10:**
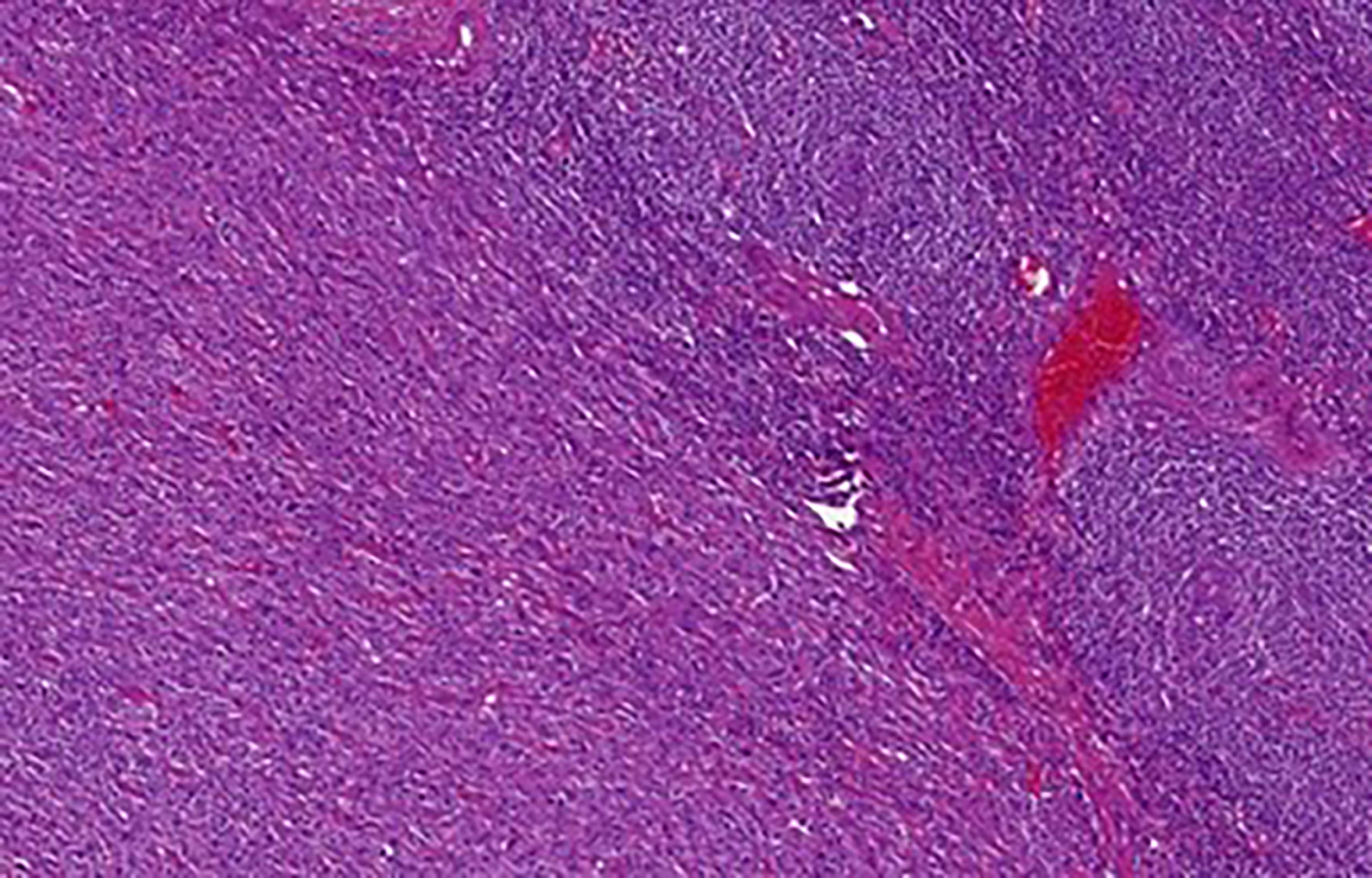
Castleman disease, hyaline-vascular type with transformation into follicular dendritic cell sarcoma: Abnormal follicles with concentric layering of lymphocytes in the mantle zone are evident in the upper right. The bottom left part of the image shows transformation into follicular dendritic cell sarcoma. The tumor cells were diffusely CD35 and CD21 positive (not shown).

The differential diagnosis of UCD is broad, ranging from common benign localized lymphadenopathy to a malignant disease such as lymphoma. It is also very important to distinguish between the UCD and the progression of a multicentric form. Laboratory tests are very helpful in this regard. While in UCD laboratory tests are usually normal, abnormalities are usually present in MCD such as CBC + differential, biochemistry, albumin, total protein, elektrophoresis, polyclonal immunoglobulins, iron, ferritin, interleukin 6, basic urine test. These abnormalities can be caused by the progression of the multicentric form or possible association with other hematologic diseases such as myeloma, lymphoma, amyloidosis or POEMS syndrome (16). Transformation of Castleman disease into a follicular dendritic cell sarcoma must be ruled out as well (**Figure 10**).

### Therapy

The optimal treatment of localized UCD is surgical removal, although this is not always entirely possible, mostly due to the complicated anatomical region affected. A lot of authors have focused on their own experience or on the literature data and concluded the surgical solution is effective, radical and safe with low recurrence rate (6–9). The surgical procedure can be performed by open or minimally invasive techniques (laparoscopy, thoracoscopy). There are also first reports of a robotic resection (17). Highly vascularized lesions can be embolized preoperatively. Especially for large masses, preoperative embolization is very helpful. This is a relatively easy procedure and it may reduce peroperative blood loss. The complexity of the surgical procedure depends on the location and size of the mass. It can be an uncomplicated straightforward procedure as well as a very demanding surgery. During the operation we must always be prepared to decide whether it is feasible to remove the whole tumor radically without compromising any of the important structures. At the same time, we have to keep in mind that we are dealing with a benign disease. For these reasons, in our opinion, it is optimal to have a biopsy result prior to the surgery (see above).

In unresectable lesions partial resection is also indicated followed by adjuvant radio or chemotherapy. Most common chemotherapy consists of anti-interleukin 6 (siltuximab) or anti-CD20 (rituximab) (18). The indication for the radiotherapy must be considered very carefully according to the localization of the mass, as its side effects on certain vital structures can be especially harmful. In some cases, a conservative approach (watch-and-wait) is a viable option. This strategy should be altered upon clear signs of progression of the disease. All these decisions must be considered by an experienced multidisciplinary team.

### Malignant potential

Secondary malignancies are not uncommon. There is an increased risk of developing follicular dendritic cell sarcomas (**Figure 10**) and Hodgkin or non-Hodgkin lymphomas in UCD (8). Therefore, these patients must be followed by a specialist for a long time.

## Discussion

Castleman´s disease is a very heterogenous group of disorders. In general, it´s a proliferation of a single or multiple lymph nodes. In the past, certain synonyms were used such as follicular lymphoreticuloma, giant cell lymph node hyperplasia or benign giant lymphoma. Aetiology and pathogenesis of CD is different for each particular type of this heterogeneous disease. The real incidence is not very well known and the male to female ratio is approximately equal. No race predominance has been observed (3, 4). After exclusion of immunodeficiencies such as an HIV patients organ transplant patients or long lasting corticotherapy, no other specific risk factors for UCD have been observed. Castleman´s disease is classified into 2 basic types according to the pathological findings - UCD and MCD.

MCD is characterized by a generalized lymphadenopathy accompanied by other severe clinical symptoms and a has higher potential for malignant transformation (6). The diagnosis of MCD sometimes presents a clinical challenge and may be delayed. On the other hand, UCD typically manifests as a proliferation of a single or multiple lymph nodes in one anatomic region and is usually asymptomatic. Patients are diagnosed with an atypical tumor of an unknown origin. The surgeons often aim for a complete resection without knowing the biological nature of the lesion prior to the operation. However, this can lead to indication errors during the operation. The complete surgical resection is of course the best primary treatment modality for UCD with excellent long-term survival and low recurrence rates (5). However, the potential for complete resection is not always clear preoperatively, and must be discussed between a surgical specialist, radiologist, oncologist etc. One of the crucial pieces of information is therefore a preoperative knowledge of “tumor histology”. Only then can we make the right decision regarding our surgical strategy. Consider the following example. If the mass is localized in the retroperitoneum, oftentimes the perirenal fat or even the kidney capsule is also affected. A completely different approach must be adopted if we assume the lesion to be a malignant tumor (a soft tissue sarcoma, metastasis of a seminoma, urogenital carcinoma etc.) rather than Castleman´s disease. In such cases, radical tumor removal with a nephrectomy is indicated in most cases. A nephrectomy with a late definitive histological result of UCD will cause harm to the patient. The same situation can occur if the tumor adheres firmly to a part of the gastrointestinal tract (small bowel, large bowel, liver, pancreas). In cases where a preoperative biopsy is for various reasons impossible, an experienced radio-diagnostician can determine the diagnosis with a very high probability using the above- mentioned imaging methods.

If the UCD is unresectable, mostly due to its size or location, conservative approach is indicated. Asymptomatic UCD may be observed, symptomatic variants should be treated with chemotherapy (rituximab, anti-interleukin-6 antibody) or in selected cases with radiotherapy (9).

Finally, we strongly believe that patients with Castleman´s disease should be treated in the high- volume centers, with a greater experience in major surgical procedures as well as with available preoperative imaging diagnostic techniques. Specialized pathologists and oncologists focusing on this issue are also absolutely necessary to avoid misdiagnosis (for example transformation of UCD into follicular dendritic cell sarcoma). Only this complex multidisciplinary approach can lead to excellent outcomes in patients with UCD. Anyone interested in this issue can focus on Castleman Disease Collaborative Network (CDCN) guidelines which are free available (19).

## Data availability statement

The raw data supporting the conclusions of this article will be made available by the authors, without undue reservation.

## Author contributions

All authors listed have made a substantial, direct, and intellectual contribution to the work and approved it for publication.
